# Cerebral glucose metabolism and Cerebral blood flow in thyroid dysfunction: An Activation Likelihood Estimation Meta-analysis

**DOI:** 10.1038/s41598-020-58255-5

**Published:** 2020-01-28

**Authors:** Kyoungjune Pak, Mijin Kim, Keunyoung Kim, Bo Hyun Kim, Seong-Jang Kim, In Joo Kim

**Affiliations:** 1 Department of Nuclear Medicine and Biomedical Research Institute, Pusan National University Hospital, Busan, Republic of Korea; 2 Department of Internal Medicine and Biomedical Research Institute, Pusan National University Hospital, Busan, Republic of Korea; 3 Department of Nuclear Medicine and Research Institute for Convergence of Biomedical Science and Technology, Pusan National University Yangsan Hospital, Yangsan, Republic of Korea

**Keywords:** Molecular neuroscience, Molecular neuroscience, Thyroid diseases, Thyroid diseases

## Abstract

Thyroid dysfunction is frequently associated with functional disturbances of the brain. We performed a meta-analysis of previous positron emission tomography and single-photon emission computed tomography studies using a coordinate-based technique of activation-likelihood estimation (ALE) to investigate the potential background of neuropsychiatric complications in patients with hypo- and hyperthyroidism. We performed a systematic search of MEDLINE and EMBASE for English-language publications using the keywords of “positron emission tomography”, “single-photon emission computed tomography”, and “thyroid”. The software GingerALE ver 2.3.6 was used to transform all reported coordinates into stereotactic Montreal Neurological Institute space. A threshold of uncorrected p < 0.001 (minimum volume of 200 mm^3^) was applied to the resulting ALE map using cerebral metabolic rate of glucose (CMRglu), and cerebral blood flow (CBF). Six studies were eligible for inclusion in the study; 4 studies of cerebral metabolic rate of CMRglu, and 2 studies of CBF. In hypothyroidism, significant decreases in CMRglu were identified in 3 clusters including left anterior cingulate, right inferior occipital gyrus, and right cuneus. In hyperthyroidism, a significant decrease in CMRglu was identified in right superior frontal gyrus. In hypothyroidism, a significant decrease in CBF was observed in left postcentral gyrus. In conclusion, several brain regions showed altered CMRglu and CBF in patients with thyroid dysfunction compared with euthyroid controls. These findings might account for underlying mechanisms of thyroid hormones on psychological and physiological effects on brain.

## Introduction

Thyroid dysfunction is frequently associated with functional disturbances of the brain such as cognitive impairment^1^, neurodegenerative disorders^2^, dementia^3^, depression, and anxiety^4^. Transient thyroid dysfunction may also induce neuropsychiatric changes^5^. These disorders accompanying thyroid dysfunction are generally reversible after return to the euthyroid status^6,7^. Therefore, it seems that mood and cognitive impairments are often associated with putative disturbance of thyroid metabolism in the brain^8^. In spite of advances in the understanding of the metabolism and action of thyroid hormone in the human brain, the relationships between these neuropsychiatric disorders and brain metabolic function are poorly understood. Therefore, it is important to increase our understanding of the pathophysiology of neuropsychiatric disorders in patients with hypo- and hyperthyroidism and to translate these findings into more effective approaches for prevention and treatment.

Although there is no direct method to measure brain function, functional neuroimaging techniques of positron emission tomography (PET) and single-photon emission computed tomography (SPECT) have provided some promising insights into the underlying mechanisms of action of thyroid hormone on cerebral metabolic rate of glucose (CMRglu) and cerebral blood flow (CBF)^8^. Previously, patients with hypothyroidism have shown a global decrease in CMRglu and CBF^9–11^, especially in posterior regions^10,11^ or in the parietal lobe^9^. Diffuse metabolic and perfusion deficits have also been observed in patient with hyperthyroidism^12–14^. However, neuroimaging studies of thyroid dysfunction have yielded inconsistent findings^9–14^.

Hence, we performed a meta-analysis of previous PET and SPECT studies using a coordinate-based technique of activation-likelihood estimation (ALE) to investigate the potential background of neuropsychiatric complications in patients with hypo- and hyperthyroidism.

## Materials and Methods

### Data search, study selection, and data extraction

We performed a systematic search of MEDLINE (from inception to 26th December 2018) and EMBASE (from inception to 26th December 2018) for English-language publications using the keywords of “positron emission tomography”, “single-photon emission computed tomography”, and “thyroid”. The inclusion criteria were original research articles that reported the difference in cerebral glucose metabolism or cerebral blood flow in subjects with hyper/hypothyroidism. Results that reported as coordinates in a normalized standard stereotactic space (Talairach or Montreal Neurological Institute space) were included. Studies based on regions-of-interest were excluded. Data were extracted from the publications independently by two reviewers, and the following information was recorded: year of publication, country of affiliations of corresponding authors, name of journal, number of studies included, database search, radiopharmaceuticals, coordinates, number of subjects included. Data sharing is not applicable to this article as no new data were created or analyzed in this study.

### Meta-analysis algorithm

The software GingerALE ver 2.3.6 (Research Imaging Institute, University of Texas Health Science Center at San Antonio, TX, USA) was used to transform all reported coordinates into stereotactic Montreal Neurological Institute space. The method used in this study is a variation of an original ALE by Turkeltaub *et al*.^15^, and later modified by Eickhoff *et al*.^16,17^. For each experiment, the modeled activation map is calculated by finding the maximum across each focus’s Gaussian. On the basis of empiric estimates of between-subject variability from the number of subjects in each study, the width of the Gaussian probability distribution is determined individually for each experiment. For each voxel, ALE value is calculated form the union of the modeled activation map. ALE values were combined across studies and tested against a null hypothesis of random distribution of ALE values, which are higher than could be expected by chance. A threshold of uncorrected p < 0.001 (minimum volume of 200 mm^3^) was applied to the resulting ALE map. ALE results were overlaid onto an anatomical template using Mango ver 4.0.1 (Research Imaging Institute, University of Texas Health Science Center at San Antonio, TX, USA).

## Results

### Literature search and study characteristics

**Figure 1:**
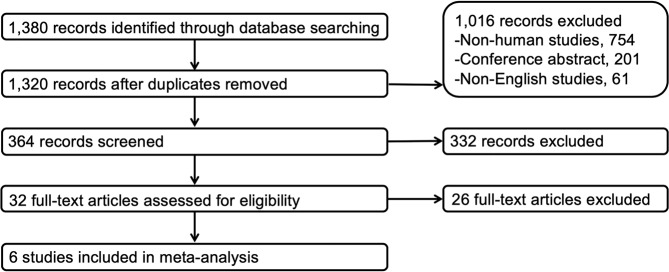
Flowchart for the identification of eligible studies.

The electronic search identified 1,380 articles. 1,348 studies that did not meet the inclusion criteria based on their title and abstract were excluded (duplicates 60, non-human studies 754, conference abstract 201, non-English studies 61). After reviewing abstracts of 364 studies, 332 records were excluded. After reviewing the full-text of 32 articles, 6 studies were eligible for inclusion in the study. Four studies of CMRglu^5,13,14,18^, and 2 studies of CBF^10,19^ were included. The detailed procedure is shown in Fig. 1. In 4 studies^10,13,14,18^, subjects of hypothyroidism/hyperthyroidism were compared with controls of euthyroid state. In a study by Jeong *et al*.^5^, 20 patients with thyroid carcinoma who underwent total thyroidectomy and discontinued levothyroxine therapy before radioactive iodine ablation (hypothyroid state) were compared with 20 patients who underwent total thyroidectomy and continued levothyroxine therapy (euthyroid state). In a study by Schraml *et al*.^19^, 9 patients with thyroid carcinoma who underwent total thyroidectomy and discontinued levothyroxine therapy for radioactive iodine ablation (hypothyroid state) were compared with each patient after thyroid hormone replacement. Study characteristics are summarized in Table 1.

**Table 1:** Studies included in this meta-analysis.

Category	Author	Year	Country	Radiopharmaceuticals	Scanner	No. of subjects	
						Patients (females)	Control (females)
**Cerebral glucose metabolism**							
Hypothyroidism							
	Jeong *et al*.	2017	Korea	^18^F-FDG	PET	20 (12)	20 (18)
	Bauer *et al*.	2009	USA	^18^F-FDG	PET	14 (11)	10 (8)
Hyperthyroidism							
	Miao *et al*.	2011	China	^18^F-FDG	PET	10 (5)	20 (10)
	Schreckenberger *et al*.	2006	Germany	^18^F-FDG	PET	12 (10)	20 (?)
**Cerebral blood flow**							
Hypothyroidism							
	Schraml *et al*.	2006	USA	^99m^Tc-ECD	SPECT	9 (4)	9 (4)
	Krausz *et al*.	2004	Israel	^99m^Tc-HMPAO	SPECT	10 (10)	10 (9)

PET, positron emission tomography; SPECT, single-photon emission computed tomography.

### Anatomical likelihood estimate analysis

#### Cerebral glucose metabolism

**Figure 2:**
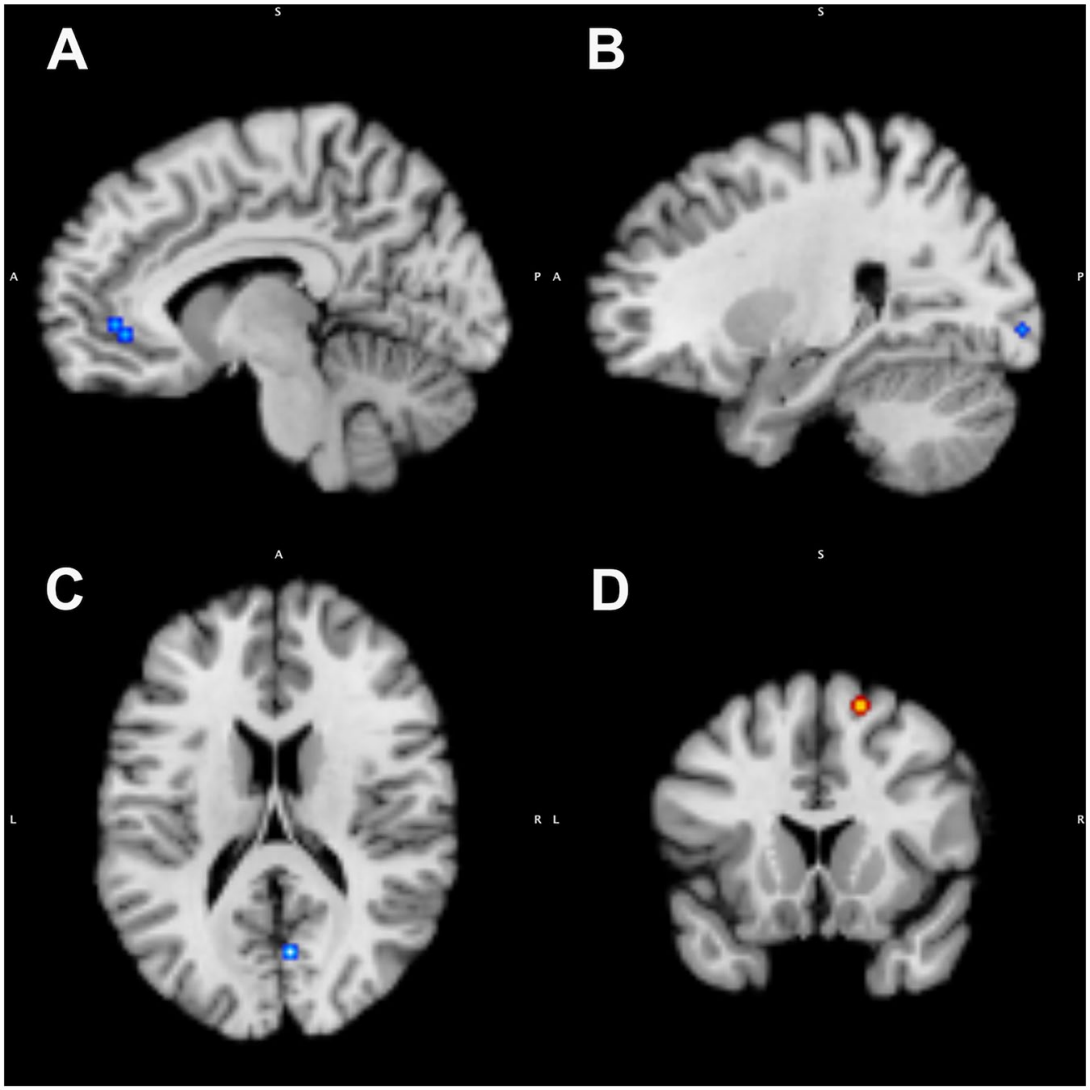
ALE analysis of CMRglu; (1) hypothyroidism < control (A, left anterior cingulate; B, right inferior occipital gyrus; C, right cuneus), and (2) hyperthyroidism > control (D, right superior frontal gyrus).

In hypothyroidism, significant decreases in CMRglu were identified in 3 clusters including left anterior cingulate (cluster size 296 mm^3^, x −8; y 44; z −6, maximum ALE value of 0.0088), right inferior occipital gyrus (cluster size 216 mm^3^, x 26; y −100; z −6, maximum ALE value of 0.0096), and right cuneus (cluster size 216 mm^3^, x 6; y −72; z 14, maximum ALE value of 0.0096). In hyperthyroidism, a significant decrease in CMRglu was identified in 1 cluster of right superior frontal gyrus (cluster size 200 mm^3^, x 16.9; y 15.6; z 60.7, maximum ALE value of 0.0085) (Fig. 2 & Table 2).

**Table 2:** Anatomical likelihood estimate analysis of Cerebral glucose metabolism.

Region	Hemisphere	Brodmann area	Volume (mm^3^)	Weighted center in MNI space			Maximum ALE value
				*x*	*y*	*z*	
**Hypothyroidism < Control**							
Anterior cingulate	L		296	−8	44	−6	0.0088
Inferior occipital gyrus	R	17	216	26	−100	−6	0.0096
Cuneus	R	30	216	6	−72	14	0.0096
**Hyperthyroidism < Control**							
Superior frontal gyrus	R	6	200	16.9	15.6	60.7	0.0085

MNI, Montreal Neurological Institute; ALE, activation-likelihood estimation.

#### Cerebral blood flow

**Figure 3:**
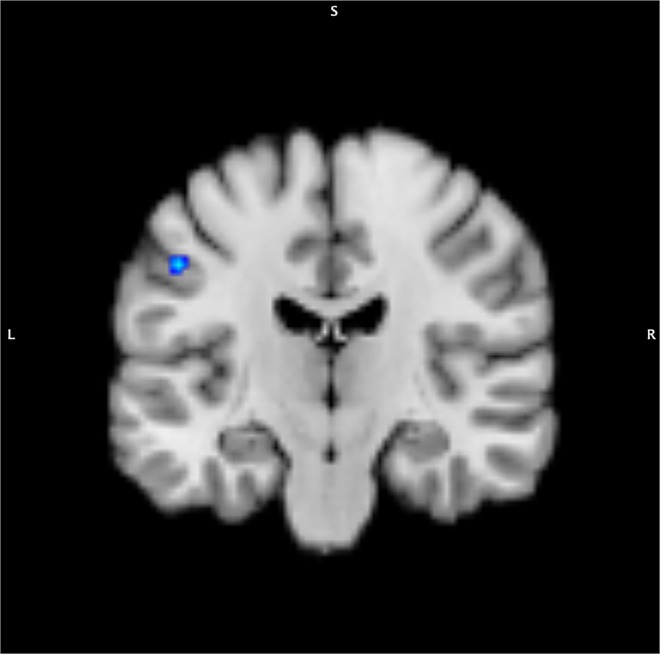
ALE analysis of CBF; hypothyroidism < control (left postcentral gyrus).

In hypothyroidism, a significant decrease in CBF was observed in 1 cluster of left postcentral gyrus (cluster size 368 mm^3^, x −53; y −15; z 41, maximum ALE value of 0.0087) (Fig. 3 & Table 3).

**Table 3:** Anatomical likelihood estimate analysis of Cerebral blood flow.

Region	Hemisphere	Brodmann area	Volume (mm^3^)	Weighted center in MNI space			Maximum ALE value
				*x*	*y*	*z*	
**Hypothyroidism < Control**							
Postcentral gyrus	L	3	368	−53	−15	41	0.0087

MNI, Montreal Neurological Institute; ALE, activation-likelihood estimation.

## Discussion

In this meta-analysis, we identified several brain regions in which patients with thyroid dysfunction reliably exhibited metabolic or perfusion decrease compared with euthyroid controls. The CMRglu in left anterior cingulate, right inferior occipital gyrus, and right cuneus was affected in hypothyroid patients. In addition, in hypothyroid status, CBF was also significantly decreased in left postcentral gyrus. On the other hand, in patients with hyperthyroidism, a significant decrease in CMRglu was identified in right superior frontal gyrus. These results suggest that thyroid hormone regulates regional CMRglu and CBF in the mature brain. These findings also demonstrate that the mechanism of neuropsychiatric disturbances in patients with hypothyroidism differs from those with hyperthyroidism.

Brain is the main target organ of thyroid hormones, and adult-onset thyroid dysfunction can have a significant impact on neuropsychiatric disturbances^1–5^. In patients with thyroid dysfunction, there may be behavioural abnormalities that mimic depression, mania, and dementia due to hormonal excess and deficit^8^. The thyroxine is a prohormone and converted to triiodothyronine (T3) within cells via deiodinase enzymes^20^. Regulation of T3 production by expression of brain-region specific deiodinase, thyroid hormone transporters and receptors are believed to maintain thyroid hormone homeostasis in the brain^21–23^. Therefore, current laboratory tests forthyroid dysfunction may not accurately measure thyroid hormone status in the brain^8,21–23^. Functional neuroimaging studies suggest a direct association between thyroid and brain activity, therefore, these can provide some clues of underlying mechanisms of thyroid hormones on psychological and physiological effects on brain.

The anterior cingulate cortex and the parieto-occipital area including cuneus and postcentral gyrus showed decreased CMRglu and CBF in patients with hypothyroidism. The anterior cingulate cortex plays an important role in affective and cognitive regulation, involving attention, problem solving, motivation, error detection, decision making, and social behaviors^24,25^. In a study of 13 hypothyroid patients and 10 euthyroid controls, hypothyroid patients exhibited lower CMRglu in anterior cingulate cortex^18^. Thyroid hormone replacement therapy has caused a reduction in the somatic complaints and depressive symptoms associated with a restoration of metabolic activity in the brain^18^. Another study identified reversible hypoperfusion in the anterior and posterior cingulate cortex, amygdala and hippocampus in previously untreated hypothyroidism^26^. Similarly, decreased pattern of brain metabolism in parieto-occipital areas was consistent with most previous neuroimaging studies performed on hypothyroid patients^9,10,19,27^. Metabolic and perfusion deficits in these area can affect working memory and attention, written word recognition, transient memory retrieval, awareness and imagery of visuospatial input, and priming processes, often compromised in patients with hypothyroidism^28^. Therefore, it is noteworthy that the anterior cingulate cortex and parieto-occipital area were identified as regions of decreased brain metabolism in this ALE meta-analysis.

In patients with hyperthyroidism, a significant decrease in CMRglu was identified in superior frontal gyrus of the right hemisphere. Miao *et al*.^13^ showed a decreased CMRglu in the left parahippocampal, fusiform, and right superior frontal gyrus, when comparing hyperthyroid patients with euthyroid controls. And, treatment with methimazole specifically increased regional activity in these regions and these changes significantly correlated with the anxiety and depressive symptoms^13^. In another included study, the CMRglu in the limbic system is affected in hyperthyroid subjects with significantly correlated with both anxiety and depressive symptoms^14^. The superior frontal gyrus is located at the superior part of the prefrontal cortex and several studies have found a broader role of this region in anatomical and functional connectivity^29^. The functional analysis demonstrates that the superior frontal gyrus has positive association with cingulate cortex, middle frontal gyrus, caudate, and thalamus and negative association with inferior frontal and precentral gyrus^30^. Further research on the value of superior frontal gyrus in mood changes caused by hyperthyroidism is needed to support our findings.

This study has some limitations. The small number of studies included in this meta-analysis meant that we had relatively limited power to detect brain regions with significance. However, we have minimized selection bias by excluding the studies based on the region-of-interest. The region-of-interest approach might result in a targeted but biased search for metabolic abnormalities. Also, we could not perform subgroup analysis based on the sex difference due to insufficient data. In addition, this study cannot demonstrate the specific relationship between altered CMRglu or CBF and clinical symptoms. Nevertheless, this meta-analysis is the first to evaluate the potential background of neuropsychiatric complications in patients with hypo- and hyperthyroidism. In addition, there was no previous study that reported no significant difference of CMRglu and CBF in hyperthyroidism and hypothyroidism. However, publication bias may exist in these previous studies.

In conclusion, brain regions with altered CMRglu and CBF were observed in patients with thyroid dysfunction. These particular regions differ in patients with hypothyroidism from those with hyperthyroidism. These findings might account for underlying mechanisms of thyroid hormones on psychological and physiological effects on brain. Further study is needed to valid the relationship between change of CMRglu and/or CBF in these regions and neuropsychiatric disturbances in the future.
