# Author Correction: Cerebral glucose metabolism and Cerebral blood flow in thyroid dysfunction: An Activation Likelihood Estimation Meta-analysis

**DOI:** 10.1038/s41598-020-62219-0

**Published:** 2020-03-19

**Authors:** Kyoungjune Pak, Mijin Kim, Keunyoung Kim, Bo Hyun Kim, Seong-Jang Kim, In Joo Kim

**Affiliations:** 1 Department of Nuclear Medicine and Biomedical Research Institute, Pusan National University Hospital, Busan, Republic of Korea; 2 Department of Internal Medicine and Biomedical Research Institute, Pusan National University Hospital, Busan, Republic of Korea; 3 Department of Nuclear Medicine and Research Institute for Convergence of Biomedical Science and Technology, Pusan National University Yangsan Hospital, Yangsan, Republic of Korea

Correction to: *Scientific Reports* 10.1038/s41598-020-58255-5, published online 28 January 2020

In the original PDF version of this Article, Seong Jang Kim and In Joo Kim were incorrectly listed as corresponding authors.

Additionally, Mijin Kim was omitted as a corresponding author. Correspondence and request for materials should be addressed to ilikechopin@me.com and mijinkim08@gmail.com. These errors have now been corrected in the HTML and PDF versions of the Article.

